# Risk Factors for Neonatal Mortality in Preterm Newborns in The Extreme South of Brazil

**DOI:** 10.1038/s41598-020-64357-x

**Published:** 2020-04-29

**Authors:** Marcos Roberto Tietzmann, Pedro do Valle Teichmann, Cassia Simeão Vilanova, Marcelo Zubaran Goldani, Clécio Homrich da Silva

**Affiliations:** 1 Graduate Program in Child and Adolescent Health, Faculdade de Medicina – Universidade Federal do Rio Grande do Sul, Porto Alegre, RS 90035-003 Brazil; 2 Faculdade de Medicina – Universidade Federal do Rio Grande do Sul, Porto Alegre, RS 90035-003 Brazil; 3 Pediatric Service, Hospital de Clínicas de Porto Alegre, Porto Alegre, RS 90035-007 Brazil; 4 Department of Pediatrics, Universidade Federal do Rio Grande do Sul, Porto Alegre, RS 90035-003 Brazil

**Keywords:** Neonatology, Preterm birth

## Abstract

Neonatal mortality still remains a complex challenge to be addressed. In Brazil, 60% of neonatal deaths occur among preterm infants with a gestational age of 32 weeks or less (≤32w). The aim of this study was to evaluate the factors involved in the high mortality rates among newborns with a gestational age ≤32w in a socioeconomically developed southern city in Brazil. Data on retrospective births and deaths (2000–2014) were analyzed from two official Brazilian national databases. The risk of neonatal death for all independent variables (mother’s age and schooling, prenatal visits, birth hospital, delivery method, gestational age, and the newborn’s sex, age, and birth year, gemelarity, congenital anomalies and birthplace) was assessed with a univariable and a multivariable model of Cox’s semiparametric proportional hazards regression (p < 0.05). Data of 288,904 newborns were included, being 4,514 with a gestational age ≤32w. The proportion of these early newborns remained stable among all births, while the neonatal mortality rate for this group tended to decrease (p < 0.001). The adjusted risk was significantly for lower birthweight infants (mean 659.13 g) born from Caesarean (HR 0.58 [95% CI 0.47–0.71]), but it was significantly higher for heavier birth weight infants (mean 2,087.79) also born via Caesarean section (HR 3.71 [95% CI 1.5–9.15]). Newborns with lower weight seemed to benefit most from Cesarean deliveries. Effort towards reducing unacceptably high surgical deliveries must take into account cases that the operations may be lifesaving for mother and/or the baby.

## Introduction

The reduction in infant mortality rates over the past few years has enabled Brazil to attain the fourth goal of the eight Millennium Development Goals proposed by the United Nations: to reduce infant mortality by two-thirds between 1990 and 2015^1^. However, unlike postneonatal mortality, neonatal mortality has not yet reached acceptable rates considering the country’s technological and economic development. Neonatal mortality in Brazil still remains a complex challenge to be addressed^2^.

Most neonatal deaths occur among preterm newborns^3^. In Brazil, 60% of neonatal deaths occur among preterm infants with a gestational age of 32 weeks or less^4^. Of all neonatal deaths, 75% occurred between zero and six days of age, and one in four deaths occurred during the first 24 hours of life^1,5,6^. Despite this scenario, there are few studies that seek to elucidate the causes or factors contributing to mortality in this population.

This study investigated the possible contributing factors involved in the high mortality rates among newborns with a gestational age of up to 32 weeks at a socioeconomically developed capital city in southern Brazil. Data were obtained through the vital statistics published by two official health information systems combined by means of a record linkage in a time series.

## Methods

Porto Alegre is the capital city of the state of Rio Grande do Sul, Brazil, with a population of 1,467,823 (2013) and a very high Human Development Index (0.805). Almost all births (99%) in Porto Alegre take place within the municipality in one of its ten hospitals (three of them university hospitals) that contain a Neonatal Intensive Care Unit (NICU). These units are also reference centers for high-risk pregnancies at both metropolitan and state level^7^. The municipality’s vital statistics (births and deaths) are processed by the Live Birth Information System (*Sistema de Informações de Nascidos Vivos* - SINASC) and by the Mortality Information System (*Sistema de Informações de Mortalidade* - SIM) and integrate a high-quality data set^6^.

This study used data from birth and death certificates of infants under 1 year old issued between 2000 and 2015. The data were obtained from the SINASC and SIM systems with the assistance of the General Coordinating Committee for Health Vigilance of the Municipal Health Office of Porto Alegre. Record linkage between the databases was performed using a deterministic procedure on STATA®. We retrieved the Live Birth Declaration numbers from both systems and resorted to a manual linkage procedure when necessary.

Newborns weighing less than 500 g were excluded from the study to minimize inclusion of births where survival was unfeasible^8,9^. The records were divided in three groups: term births (infants born at a gestational age of 37 weeks or more), late preterm births (infants born at a gestational age between 32 and 36 weeks and 6 days), and early preterm births (infants born at a gestational age of less than 32 weeks). Only the records of early preterm newborns were included in the multivariable analysis. Infants without a death certificate and those who died after the 27th day were considered survivors. The independent variables used were the mother’s age and schooling, number of prenatal visits, type of hospital where the birth took place, delivery method, gestational age, and the newborn’s sex, age, and birth year, gemelarity, congenital anomalies and outhospital birth. Dependent variables were death and age of death.

Gestational ages on the birth certificates from 2000–2010 were classified into three categories relevant to this study: (1) less than 22 weeks; (2) 22–27 weeks; and (3) 28–31 weeks^10^. Even though these continuous variables sometimes extended days or weeks into 2011, these categories were kept based on the previous criteria. Conforming previous study with data from 2011 year, the preferential information padronized for gestational age colected was last menstrual period, and alternatively accepted direct information from physical exam or other methods. Last menstrual period was informed in 42,2% of birth registrys and other methods or method not informed in 54%. No gestational age information or missing cases added up 3,8%^10^. Over time the completeness of SINASC information is improving for most of variables, including duration of gestation^11^.

The National Registry of Health Facilities of the Brazilian Ministry of Health classifies hospitals into three basic categories: (1) public hospitals exclusively funded by the national public health system (*Sistema Único de Saúde* - SUS); (2) private hospitals funded by private health insurance plans and the patients’ own funds; and (3) mixed hospitals (non-profit private institutions) funded by SUS, private health insurance plans or by the patients’ own funds. For analysis purposes, this study considered private and mixed hospitals as one single category (private).

First, we performed a descriptive analysis of all variables. In multivariable modelling, the conceptual model was from farthest to the nearest for outcomes. Birthweight was also analyzed both as a continuous and categorical variable (in quintiles) to check for model adequacy and interactions with the other variables. Birth year was also included in the final model to control for general changes in risk of death throughout the years and to allow for a time evolution of the mortality outcome.

We then performed a survival analysis of the early newborn group using a univariable and a multivariable model of Cox’s semiparametric proportional hazards regression, calculated with the Survival package included with the R software^12^. Were considered statistically significant *p* values <0.05. Each covariate was individually assessed with Kaplan-Meier curve analysis and Schoenfeld residuals. They were then assessed together through a global correlation between covariates and time. Both procedures indicated that the data were adequate for Cox’s proportional hazards regression^13^.

For statistical modelling, we performed a univariable regression analysis of each independent variable for the time to event outcome (death or survival), then investigated their interactions. Those with a statistically significant difference were used in the next phase, which applied the multivariable model with the technique known as “backward stepwise”. The final main model was chosen by comparing the likelihood ratio tests (“goodness-of-fit”).

The consistency of the model was assessed based on its application to different scenarios composed by database variables containing only: a) deliveries in private hospitals; b) deliveries in public hospitals; c) deliveries by mothers with less than eight years of schooling; and d) deliveries between 2006 and 2014. The final model was used on the dataset on early newborns (gestational age <32 weeks) and separately on each weight quintile, adding up to a total of six regressions.

This study was approved by the Research Ethics Committee of the Hospital de Clínicas de Porto Alegre (protocol no. 17/0124) and by the Municipal Health Office of Porto Alegre (certificate no. 60897216.4.0000.5327). As the present research used secondary data from the municipality’s health information systems, the informed consent form was waived. Thus, all methods were performed in accordance with the latest current guidelines and regulations of the National Health Council of the Brazilian Ministry of Health (Resolution No. 466/2012 and No. 580/2018).

## Results

We analyzed 288,904 records issued between 2000 and 2014 (Table 1). The chi-square test for trend showed that the proportion of early preterm births remained stable, while the neonatal mortality rate for this group tended to decrease (*p* < 0.001). A total of 1,845 infants died during the first 27 days of life, with 21% of deaths occurring on the birthday, 45% between days 1 and 6, and 33% between days 7 and 27.

**Table 1:** Births and neonatal mortality trends from 2000–2014 according to gestational age groups in Porto Alegre, Brazil.

Year	*n*		Early preterm births		Late preterm births		Term births	
			(GA 22–31weeks)		(GA 32–36 weeks)		(GA ≥ 37 weeks)	
	Live births	NNM	Live births (%)	NNM	Live births (%)	NNM	Live births (%)	NNM
2000–2002	63,925	7.6	1.47	4.0	8.46	1.4	89.86	1.9
2003–2005	57,199	6.9	1.54	3.7	9.04	1.3	89.37	1.8
2006–2008	54,351	6.3	1.58	3.3	9.24	1.1	89.12	1.9
2009–2011	55,497	5.5	1.66	3.0	9.96	1.0	88.33	1.3
2012–2014	57,932	5.2	1.55	2.6	9.57	1.0	88.80	1.5
P^a^	—	1,39E-06	0.07688	3,82E-03	<2.2e-16	0.009521	3,36E-12	0.01244

GA Gestational Age.

NNM Neonatal Mortality.

NMA Number of neonatal deaths/number of newborns x 1000, GA Gestational age.

^a^P-value of Chi-squared Test for Trend in Proportions.

**Figure 1:**
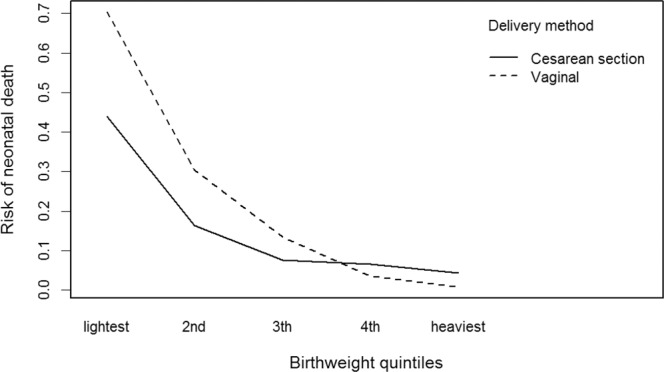
Interaction between delivery method and birthweight.

With early preterm group records, we analyzed 4,514 newborns. Cox’s univariable semiparametric regression analysis showed all variables to have statistical significance except gemelarity (Table 2). After adjustment for the other variables, maternal age and schooling did not have any statistical significance, while attending less than four prenatal care visits was considered a risk for neonatal death compared to four or more visits (HR 1.21 [95% CI 1.05–1.40]). Deliveries in public hospitals had a greater risk for neonatal mortality compared to deliveries in private hospitals (HR 1.54 [95% CI 1.33–1.78]). Male newborns were at greater risk of non-survival than female newborns (HR 1.39 [95% CI 1.22–1.59]). With weight as a continuous variable, the risk rate changed little between univariable and multivariable analyses, meaning that weight provided protection against neonatal mortality (HR 0.996 [95% CI 0.995–0.996]). The results of the adjusted analyses for delivery method followed two distinct directions, as there was a significant interaction between delivery method and birthweight (*p* < 0.001). Cesarean delivery generally provided greater protection against neonatal mortality compared to vaginal delivery (HR 0.19 [95% CI 0.12–0.30]), but the interaction between delivery method and birthweight showed a greater risk with Cesarean delivery depending on weight gain (HR 1.001 [95% CI 1.0009–1.0019]). As shown in Fig. 1, vaginal delivery offers less protection in the lower quintiles, but provides greater protection in the higher weight quintiles (4^th^ and 5^th^).

**Table 2:** Description analysis and hazard ratios according to the characteristics of early newborns gestational age <32 weeks), mothers, and gestational periods between 2000–2014 in Porto Alegre, Brazil.

	Livebirths N (%)	Univariable regression			Multivariable regression^a^		
		HR	95%CI	P	HR	95%CI	P
**Maternal age**							
20 to 34 years^Ref^.	2809 (62.0)	1.00	Ref.		1.00	Ref.	
Less than 20 years	799 (17.7)	1.30	1.11–1.52	0.00102	1.01	0.85–1.20	0.858095
More than 34 years	903 (20.0)	0.90	0.76–1.073	0.25068	1.10	0.92–1.32	0.271338
**Maternal schooling**							
High School graduate or +^Ref.^	1158 (25.65)	1.00	Ref.		1.00	Ref.	
Less than 8th grade	1512 (33.49)	1.53	1.28–1.82	1.92e-06	1.08	0.89–1.33	0.402286
8th through 11th grade;no diploma	1810 (40.09)	1.45	1.22–1.73	1.77e-05	1.08	0.89–1.33	0.383019
**Antenatal visits**							
4 or more ^Ref.^	2798 (61.98)	1.00	Ref.		1.00	Ref.	
Less than 4 visits	1680 (37.21)	2.00	1.76–2.27	<2e-16	1.21	1.05–1.40	0.007569
**Delivery type**							
Vaginal ^Ref.^	1843 (40.82)	1.00	Ref.		1.00	Ref.	
Cesarean	2669 (59.12)	0.47	0.41–0.53	<2e-16	0.19	0.12–0.30	8.05e-12
Birthweight:cesarean interaction		—	—	—	1.001	1.0009–1.0019	2.27e-07
**Hospital type**							
Private insurance accept ^Ref.^	2148 (47.58)	1.00	Ref.		1.00	Ref.	
Public insurance only	2177 (48.22)	2.01	1.76–2.31	<2e-16	1.54	1.33–1.78	5.23e-09
**Sex**							
Female ^Ref.^	2139 (47.38)	1.00	Ref.		1.00	Ref.	
Male	2369 (52.48)	1.23	1.08–1.4	0.0012	1.39	1.22–1.59	7.91e-07
**Birth year**							
2000–02	943 (20.89)	1.71	1.40–2.09	1.36e-07	1.49	1.19–1.85	0.000334
2003–05	885 (19.61)	1.47	1.19–1.81	0.000238	1.49	1.20–1.86	0.000313
2006–08	860 (19.05)	1.26	1.01–1.56	0.034730	1.22	0.97–1.53	0.074902
2009–11	925 (20.49)	1.10	0.88–1.36	0.393218	1.17	0.93–1.47	0.156298
2012–14 ^Ref.^	901 (19.96)	1.00	Ref.		1.00	Ref.	
Birthweight (Quintile)		0.9966	0.9963–0.9968	<2e-16	0.996	0.995–0.996	<2e-16
Lighter	910 (20.16)	—	—	—	—	—	—
2th	901 (19.96)	—	—	—	—	—	—
3th	909 (20.14)	—	—	—	—	—	—
4th	913 (20.23)	—	—	—	—	—	—
Heavier	881 (19.52)	—	—	—	—	—	—
**Birth local**							
Hospital ^Ref.^	4447 (98.51)	1.00	Ref.		1.00	Ref.	
Other	65 (1.43)	2.41	1.65–3.51	4.24e-06	1.31	0.75–2.30	0.336763
**Gestational type**							
Single ^Ref.^	3748 (83.03)	1.00	Ref.		1.00	Ref.	
Multiple	764 (16.92)	0.96	0.81–1.145	0.697	0.99	0.82–1.18	0.943713
Congenital anomalies							
No	4265 (94.48)	1.00	Ref.		1.00	Ref.	
Yes	198 (4.38)	3.83	3.14–4.66	<2e-16	2.82	2.29–3.48	<2e-16
Neonatal mortality							
Survival	3537 (78.36)	—	—	—	—	—	—
Neonatal death	977 (21.64)	—	—	—	—	—	—

Livebirths are presented as number (percentage). *HR* hazard ratio, *95% CI* 95% confidence interval.

Ref. The reference group in categorical variables were the 20–34 years age group, the high school graduate or + group, the 4 or more antenatal visits group, the vaginal delivery group, the private insurance accept group, the female group, the born in the 2012–2014 period group, the born in hospital group, the single-pregnancy group and the group without congenital anomalies.

^a^Cox’s multivariable survival analysis adjusted for maternal age and schooling, number of prenatal care visits, delivery method, interaction between delivery method and birthweigth, type of hospital where the delivery took place, gestational age, gemelarity, anomalies, birthweight, sex, and birth year; Concordance = 0.837 (se = 0.01); Likelihood ratio test = 1388 on 17 df, p = 0; Wald test = 1089 on 17 df, p = 0; Score (logrank) test = 1129 on 17 df, p = 0.

Table 3 indicates greater neonatal mortality rates among infants born from vaginal delivery than from Cesarean section in the three lowest birthweight quintiles, while the opposite is seen in the highest quintile. The adjusted regression for the other variables suggests that Cesarean sections provide greater protection (HR 0.58 [95% CI 0.47–0.71]) compared to normal delivery in the lightest quintile (mean birthweight 659.13 g). In the 5^th^ quintile (mean birthweight 2,087.79), neonatal mortality becomes a risk with Cesarean births (HR 3.71 [95% CI 1.5–9.15]).

**Table 3:** Birthweight, delivery rate and neonatal survival per birthweight quintile and delivery method from 2000–2014 in Porto Alegre, Brazil.

Quintile	Total (N = 4514)	Birthweight^a^	Delivery rate		Neonatal survival			
			Vaginal	Cesarean	Univariable regression		Multivariable regression^b^	
		Mean (*sd*)	%	%	HR (*95% CI*)	P	HR (*95% CI*)	P
Lightest	910	658.14 (84.71)	54.9	45.1	0.46 (0.38–0.55)	<2e-16	0.58 (0.47–0.71)	1.45e-07
2nd	901	921.53 (69.9)	37.62	62.38	0.5 (039–0.65)	2.99e-07	0.63 (0.47–0.85)	0.00258
3th	909	1176.5 (74.16)	32.45	67.55	0.54 (0.36–0.81)	0.00296	0.57 (0.36–0.90)	0.01626
4th	913	1446.94 (89.92)	32.89	67.11	0.97 (0.55–1.71)	0.922	1.51 (0.79–2.89)	0.20863
Heaviest	881	2082.65 (530.78)	46.54	53.46	3.14 (1.36–7.27)	0.00736	3.71 (1.5–9.15)	0.004454

sd standat error, HR hazard ratio, 95% CI 95% confidence interval.

^a^Birthweight in grams^a^.

^b^Cox’s multivariable regression for delivery method, adjusted for maternal age and schooling, number of prenatal care visits, type of hospital where the delivery took place, gestational age, gemelarity, anomalies, birthweight, sex and birth year.

After applying the final model to four different scenarios (deliveries in private hospitals only, deliveries in public hospitals only, infants born to mothers with less than eight years of schooling, and infants born between 2006 and 2014, Cesarean delivery continued to be a protective factor compared to vaginal delivery for newborns with a lower birthweight (*p* < 0.05).

## Discussion

Assessment of the temporal trend for preterm birth rates showed that the proportion of early preterm births in relation to the other gestational age ranges has remained stable over the years (around 1.56% of all births), even with a percentage increase in preterm births (*p* < 0.001). The neonatal mortality rate among early preterm infants tended to decrease (*p* < 0,001), which also was observed in late preterm infants. The steady proportion of early preterm births in the context of an increase in preterm births and the difference in reduction of neonatal mortality for early preterm, late preterm, and term births may be partially explained by the preferential choices of delivery method, which is a constant subject of research^14–16^.

The descriptive analysis of the variables showed that most neonatal deaths occurred between the second and sixth day, which points to a high-quality standard of care during the first 24 hours of life. This is a favorable and positive scenario compared to the other regions of Brazil and other developing countries, where the proportion of deaths on the first day of life is often higher^1,6^. According to the multivariable regression analysis, the population most affected by neonatal mortality were congenital anomalies, low birthweight male infants, born before 2006, from vaginal delivery and at public hospitals, to mothers who had attended less than four prenatal care appointments. This rate was not associated with maternal age, schooling or gemelarity. Cesarean section was a significant factor in protecting against neonatal mortality in the lowest weight quintile. Its statistical significance decreased up to the 5th quintile, where it became a statistically significant risk for neonatal mortality.

Despite the low maternal schooling, extreme maternal age and gemelarity, three classics factors for neonatal mortality did not demonstrate a statistically significant risk after the multivariable adjustment in this study. The research conducted by Lansky *et al*. with data taken from various Brazilian states showed similar results for maternal age, although not for low schooling and gemelarity, which represented a statistically significant risk for neonatal mortality^4^.

Over the past few years, education levels, prenatal care coverage, and neonatal health care have been improving both in Porto Alegre and on a national level. This may help explain the decrease in mortality rates^1,17^. However, our results pointed to a need for qualification of prenatal care, particularly through anticipating care for pregnant women, even in socioeconomically developed cities. Early diagnosis and adequate management of high-risk pregnancies are essential to reduce the incidence of preterm births and neonatal deaths^18,19^.

Delivery at a public hospital was shown to be a risk factor for neonatal mortality. We controlled for maternal age, schooling, and number of prenatal visits in the multivariable regression, then reapplied the regression analysis to an exclusive population of mothers with eight years of schooling or less (i.e., the predominant profile in public hospitals). Public hospitals were again found to be a higher risk factor for neonatal mortality compared to private hospitals. In spite of this, there were no clinical data to support the claim that pregnant women at public hospitals were under worse health conditions than those at private hospitals. There were also no data related to perinatal care procedures such as pre-labor monitoring, corticoid use, contraction inhibitor administration, and surfactant therapy in newborns. In light of this, the significant risk of neonatal mortality associated with public hospitals may be explained by a lack of infrastructure and qualified human resources^2,20–22^. Although prenatal corticoids for pregnant women and surfactants for newborns are available in the public health system, their use is not yet widespread in the hospitals of Brazil. According to the yearly reports of the Brazilian Network on Neonatal Research comprising 20 teaching hospitals in Brazil, 60% of pregnant women who gave birth to infants under 1,500 g in 2008 had used prenatal corticoids, while 49% of these infants had received surfactant therapy. By 2016, these numbers had increased to 78% e 55%, respectively^23^.

Male newborns seemed to have a lower chance of surviving, possibly due to factors such as differences in body maturation when compared to female newborns. This subject remains under research^8,9,21,24,25^.

The risk factor represented by the delivery method changed depending on the newborn’s weight range. Cesarean sections acted as a markedly protective factor against neonatal mortality for low birthweight infants. Although our study did not rely on this particular type of clinical perinatal data, there are indications in literature that children under chronic hypoxic stress, such as fetuses with restricted intrauterine growth, could have a worse outcome following vaginal delivery due to the risk of further injury associated with perinatal hypoxia during labor^8,25^. Another risk factor associated with vaginal delivery for very early preterm newborns is breech presentation during labor. While breech presentation affects only 4% of term births, it corresponds to 20–35% of infants born at 28 weeks, which contributes to a higher mortality rate among vaginal delivery babies^26,27^. Interestingly, Cesarean section did not represent a protective factor for heavier birthweight infants, becoming in turn a significant risk factor. This may be related to a higher frequency of severe respiratory complications and use of mechanical ventilation among late preterm infants born from Cesarean sections than among those born from vaginal delivery^27,28^. Hormonal and pulmonary physiological aspects and respiratory diseases related to delivery method have also been reported in literature^29^.

Using the National Vital Statistics System of the United States, after adjusting for other risk factors, Lee and Gould found a higher death risk associated with vaginal delivery than with Cesarean section for newborns too small for gestational age (SGA) (26 to 30 weeks)^8^. According to the same study, vaginal deliveries were more protective than Cesarean sections against the same outcome for SGA newborns over 36 weeks and for appropriate-for-gestational-age (AGA) newborns over 28 weeks. Using the same database, Malloy *et al*. found Cesarean deliveries to be a protective factor for newborns with a gestational age between 22 and 25 weeks regardless of the reason behind indication for a Cesarean section^9^. The odds ratio ranged from 0.58 (95% CI: 0.38–0.87) for a gestational age of 22 weeks to 0.81 (95% CI: 0.69–0.94) for 25 weeks. Among infants born at the 31^st^ or 32^nd^ week, Cesarean delivery became a risk factor for neonatal death. These findings are consistent with the results of our study. Other studies with smaller sample sizes did not find a significant difference in risk of death among the groups born from vaginal delivery and from Cesarean sections^30–32^. Several randomized trials designed to elucidate the best preterm birth method were suspended due to difficulties in recruiting participants^27,33^.

Regarding prenatal coverage, the risk of neonatal mortality among pregnant women who had attended less than four prenatal care visits was similar to the ones found in other studies. In a neonatal survival study with data from 57 low- and middle-income countries, attending four or more prenatal visits represented a protective factor against neonatal mortality (HR 0.54 [95% CI 0.50–0.59]) compared to attending no visits^34^. Similar results were found by Dhaded *et al*.^21^.

The difference in neonatal mortality rates between different hospital types has also been observed in other studies. Lansky *et al*. analyzed 266 hospitals across all regions of Brazil in the “Nascer no Brasil” (“Being born in Brazil”) survey and found a higher mortality risk among infants born at publicly-funded hospitals (OR 2.78 [95% CI 1.37–5.6]) compared to those born at hospitals with fully private funding^4^. This study also suggested that adequate early intervention measures during situations of perinatal hypoxemia could save many lives throughout the country. This is corroborated by a previous study conducted in Belo Horizonte, the capital city of the state of Minas Gerais, also placed at the top of the Human Development Index^22^. Our study compared privately funded hospitals and mixed-fund hospitals (private and public) as one single category against publicly funded hospitals, which may have contributed to a lower risk (HR 1.54 [95% CI 1.33–1.78]). It should be noted that, despite the findings of this study, public hospitals in Porto Alegre have good ratings both on a national and international level, and most offer high-quality healthcare study programs. Further studies may lead to a clearer understanding of the risk of mortality associated with the public health system.

This is a pioneering study on population-specific risk factors for neonatal mortality in preterm infants with less than 32 weeks of gestational age. These risks have been often designated as the main cause behind the maintenance of high neonatal mortality rates in the country. Few studies in Brazil have used information systems on vital statistics to assess infant mortality.

One of the study’s limitations is in the information systems’ lack of important data for investigating outcomes, such as diseases or clinical conditions during gestation and delivery, medical treatments undergone by mothers before and after delivery, smoking habits and other factors. Likewise, gestational age was classified into categories of 22–27 weeks and 28–32 weeks, which prevents a more detailed analysis of weight adequacy by gestational age.

One of the strengths of this study is the use of two high-quality official health information systems (SINASC and SIM) recognized by the Ministry of Health and available throughout the entire national territory. Additionally, relying on a population sample allows for lower costs and a reduced execution time. Another important aspect is the use of a multivariable regression analysis of survival to obtain a better overall statistical accuracy by incorporating not only the binary neonatal mortality outcome, but also the day of the event.

Our study showed that the risk of neonatal death among early preterm infants has been declining over the years and that Cesarean delivery may be more important in reducing neonatal mortality than it is currently thought to be. The mortality risk for newborns born from vaginal delivery was shown to be significantly dependent on birthweight. Newborns with lower weight seemed to benefit most from Cesarean deliveries.

Faced with an epidemic of surgical deliveries throughout the country, the Brazilian government has been taking actions in an effort to reduce unacceptably high cesarean section rates take into account that are cases when the operations must be conducted, and make clear that many times they may lifesaving for mother and/or the baby.
